# WOME: Theory-Based Working Memory Training — A Placebo-Controlled, Double-Blind Evaluation in Older Adults

**DOI:** 10.3389/fnagi.2018.00247

**Published:** 2018-08-14

**Authors:** Juliane Weicker, Nicole Hudl, Stefan Frisch, Jöran Lepsien, Karsten Mueller, Arno Villringer, Angelika Thöne-Otto

**Affiliations:** ^1^Clinic of Cognitive Neurology, Leipzig University, Leipzig, Germany; ^2^Department of Neurology, Max Planck Institute for Human Cognitive and Brain Sciences, Leipzig, Germany; ^3^Max Planck International Research Network on Aging, Rostock, Germany; ^4^Institute of Psychology, Goethe University Frankfurt, Frankfurt, Germany; ^5^Nuclear Magnetic Resonance Unit, Max Planck Institute for Human Cognitive and Brain Sciences, Leipzig, Germany

**Keywords:** working memory training, cognitive training, plasticity, aging, neuropsychology, rehabilitation, cognitive decline, mild cognitive impairment

## Abstract

**Background:** Scientifically evaluated cognitive intervention programs are essential to meet the demands of our increasingly aging society. Currently, one of the “hottest” topics in the field is the improvement of working memory function and its potential impact on overall cognition. The present study evaluated the efficacy of *WOME* (WOrking MEmory), a theory-based working memory training program, in a double-blind, placebo-controlled, and randomized controlled trial (www.drks.de, DRKS00013162).

**Methods:** *N* = 60 healthy older adults were allocated to (1) the *WOME* intervention, (2) an active low-level intervention, or (3) a passive control group. Overall, the intervention groups practiced twelve sessions of 45 min within 4 weeks of their respective training. Transfer effects were measured via an extensive battery of neuropsychological tests and questionnaires both pre-/post-training and at a 3-month follow-up.

**Results:** *WOME* led to a significant improvement in working memory function, demonstrated on a non-trained near transfer task and on two different composite scores with moderate to large effect sizes. In addition, we found some indication of relevant impact on everyday life. The effects were short-term rather than stable, being substantially diminished at follow-up with only little evidence suggesting long-term maintenance. No transfer effects on other cognitive functions were observed.

**Conclusion:** *WOME* is an appropriate and efficient intervention specifically targeting the working memory system in healthy older adults.

**Trial Registration:** German Clinical Trials Register (DRKS), Identifier: DRKS00013162.

## Introduction

In an increasingly aging population, we would benefit from reliable research data concerning appropriate prevention and intervention treatments. Longer lifespans and aging baby boomers will lead to a dramatic rise of about one billion adults aged 65 years and older by 2030 worldwide. In addition to obvious physiological changes, aging is accompanied by declines in a range of cognitive functions including processing speed, attention, memory, reasoning, mental flexibility, and working memory (Glisky, 2007). Healthy older adults have to deal with these reductions in their mental capacity (experienced, for example, in subjective memory complaints), and it is coherent that the need for maintenance or extension of cognitive function is increasing (Fernandez, 2011). Scientifically evaluated cognitive intervention programs are therefore essential to meet the demands of our aging society.

One of the current “hot” topics in the field is the improvement of working memory (WM) function and its potential impact on overall cognition. WM, the memory system that holds contents in a temporarily accessible state, is a key predictor for successfully managing life’s demands successfully, for example, academic achievement (Gathercole et al., 2003), professional success (Higgins et al., 2007), acquisition of new skills (Pickering, 2006), and emotion regulation (Schmeichel et al., 2008). Fueled by cognitive neuroscience that discovered the plasticity of the human brain (i.e., the malleability of neuronal structure, function, and cognitive abilities), the idea emerged that by practicing WM tasks, substantial improvements could be induced in the overall WM system (Klingberg et al., 2002). In contrast to strategy based approaches, core training programs promote domain-general mechanisms which are the requirements for transfer to other tasks than the trained ones (Morrison and Chein, 2011). Given the important role of the WM capacity for many higher order cognitive functions (Baddeley, 2003), an expansion of this capacity was hypothesized to improve not only WM performance but also broader abilities, such as reasoning or intelligence (Jaeggi et al., 2008a). Indeed, multiple meta-analyses showed that intensive WM training led to significant and long-lasting improvements in overall WM functioning, and there was some indication that it transfers to associated cognitive functions (Karbach and Verhaeghen, 2014; Lampit et al., 2014; Weicker et al., 2016). Although the underlying neuronal mechanism of such transfer effects remain largely unknown, behavioral changes are found to be accompanied by transformations in brain activation, brain structure, and connectivity (for a review, see Buschkühl et al., 2012). Despite such promising findings, many studies support the assumption that age serves as a negative predictor for training benefits, especially with regard to changes beyond the WM system, which limits the potential of WM training in older adults (Brehmer et al., 2012) and some meta-analyses question the efficacy of WM training in comparison to practicing daily routines (Dougherty et al., 2016; Melby-Lervag et al., 2016). One of the main reasons for missing guidelines and recommendations arises from methodological limitations of cognitive training research (Shipstead et al., 2012; Dougherty et al., 2016). Given the nature of individually adapted treatments, which usually cannot be fully concealed from the participants, implementations of randomized, double-blind, and placebo-controlled designs are hardly feasible. While observations show cumulative implementations of randomized controlled trials over the past few years, no-contact control groups are widely used that do not account for expectancy effects (e.g., belief about performance improvements), social aspects (e.g., personal contact with laboratory assistants or other participants), or motivation (e.g., taking part in a research study; Shipstead et al., 2012). The operationalization of treatment effects is another issue, because it is necessary to demonstrate not only task-specific enhancements, but also an improvement of more general processes underlying the construct of WM function (e.g., higher capacity or cognitive control). However, many studies apply very similar tasks to evaluate WM training efficacy, and there has been doubt that the claimed transfer was valid (Melby-Lervag and Hulme, 2013). An elegant way to assess the psychological construct rather than task-specific relationships between training and transfer tasks is the analysis of multiple tests via composite scores or latent variables, which is rarely realized in WM research (Schmiedek et al., 2010; von Bastian et al., 2013; Stepankova et al., 2014; Lawlor-Savage and Goghari, 2016). Due to the complexity of intervention designs as well as the huge amount of time and effort to conduct such studies, sample sizes are usually small [e.g., the median in older adults is approximately *N* = 36 and have a high risk of producing unreliable results (Lampit et al., 2014; Weicker et al., 2016)].

The computer software industry has launched several cognitive training programs within a short time (e.g., Brain Age^®^, Lumosity^®^, NeuroNation^®^) despite the controversial conceptualization of these evaluation studies. Brain training appeals to both consumers and health economics. So-called “serious games,” easily and cheaply available on the Internet, promise a true panacea despite missing evidence (Rabipour and Raz, 2012; Federal Trade Commission, 2016). It remains rather unclear as to which task characteristics provoke transfer effects and impact the WM system successfully (Morrison and Chein, 2011). In the absence of knowledge about specific task characteristics, most of the available training programs represent a compilation of various WM tasks. The diversity of exercises increases the chance of tapping one task, or a certain combination of tasks, that successfully improves WM capacity and reduces strategy-based performance gains. Nevertheless, this “kitchen sink” approach neither reveals information about necessary components that influence the WM system nor does it refer to assumptions regarding its theoretical structure. Furthermore, many tasks that are used to train WM in young adults, for example, dual n-back tasks, are known to be inadequate or inefficient for older adults (Jaeggi et al., 2008b; Lawlor-Savage and Goghari, 2016).

The lack of a theory-based training program with specific, age-appropriate training tasks led the authors to develop an intervention that focuses on individuals with low WM abilities. The new WM training, called *WOME* (WOrking MEmory), is part of the cognitive rehabilitation software RehaCom^®^. The intervention fulfills the criteria for successful WM training postulated by Buschkühl (2007). The main principles of the program are (a) its theoretically derived structure, implemented in hierarchically ordered modules that enable targeted training of specific WM components and examine the efficacy of specific training tasks, (b) fine-tuned automatic adaptivity for preventing under challenging or over demanding task difficulty by not only adjusting the number of items to be remembered, but enable modifications of many additional task features, and (c) the implementation of everyday life stimuli to facilitate transfer effects and preserve high motivation by using age-appropriate content. The theoretical considerations were derived from Baddeley’s multi-component model of the WM system but also include insights from neurophysiological findings that emphasize the relevance of selective attention and inhibition processes in WM (Baddeley, 2003; Miyake and Shah, 2007).

The present study was designed to evaluate feasibility and efficacy of the *WOME* intervention with a solid methodological design to explore the essence of WM functioning and cognitive plasticity. Specifically, we aimed to answer the following questions: does the intervention reveal reliable effects on either the WM system or related cognitive functions? If so, do they remain stable over a long period? Are performance changes reflected in broader psychological constructs, so that factors that were affected by the intervention (e.g., specific WM components) could be identified? Are the potential cognitive improvements functionally relevant in the participants’ everyday lives? We conducted a double-blind, placebo-controlled, randomized controlled trial implementing three conditions: (1) a high-level WM training group (HT) that received the *WOME* intervention at a high level with increasing difficulty, (2) a low-level WM training group (LT) that received the same intervention, but on a low level with stable difficulty, and (3) a no-contact control group (CG). Before and after the intervention, as well as at a 3-month follow-up, we applied an extensive battery of neuropsychological tests and questionnaires to target different components of WM and related cognitive functions that require WM, as well as impact on everyday life outside a laboratory setting. Based on the presented literature, our hypothesis was that the HT would show more improvement in post-treatment performances relative to both LT and CG, in measures of WM, but only limited transfer to other cognitive domains and everyday life functions. We expected that potential benefits be preserved over the follow-up period.

## Materials and Methods

### Participants

Sixty older adults (28 male, 32 female) were selected for participation in the study according to the following inclusion criteria: (a) aged between 60 and 79 years, (b) fluent in German, (c) clinically healthy, and (d) willingness and ability to take part in an intensive training program. Exclusion criteria were (a) history of neurological and/or psychiatric disease, (b) severe cognitive deficits, (c) alcohol or drug abuse, and (d) participation in other cognitive enhancement programs. Subjects were recruited via the Institute’s database and by means of flyers distributed in the local community. The mean age of participants was 67.7 years (*SD* = 4.3, range 60–77). **Table 1** lists detailed sample characteristics for each condition. All participants gave written informed consent in accordance with the Declaration of Helsinki and were financially rewarded for participation. The study was conducted according to the CONSORT statement, approved by the ethics committee of the University of Leipzig (033-12-23012012) and registered at the German Clinical Trials Register (DRKS00013162).

**Table 1:** Sample characteristics.

Sample characteristics	HT (high-level WM training; *n* = 20)	LT (low-level WM training; *n* = 20)	CG (passive control group; *n* = 20)	Difference
Male/female	10/10	9/11	9/11	χ^2^ = 0.13, n.s.
Age (mean, *SD*)	67.8 (3.9)	67.7 (3.1)	67.5 (5.7)	*F* = 0.34, n.s.
Education level (*n*)				χ^2^ = 7.07, n.s.
<9 years	1	3	2	
10–12 years	5	11	6	
>12 years	14	6	12	
Subjective everyday life functioning rated on a 3-point Likert scale (*n*)				χ^2^ = 2.85, n.s.
No complaints	15	17	16	
Complaints not impairing everyday life	5	3	3	
Impairments in everyday life	0	0	1	
Mood (Beck Depression Inventory: mean, *SD*)	4.5 (3.2)	4.7 (2.8)	3.7 (2.9)	*F* = 0.666, n.s.
WM performance (Span Board backward: mean, *SD*)	6.5 (1.1)	7.6 (1.7)	7.2 (1.6)	*F* = 2.796, n.s.


*WM, working memory; HT, high-level WM training group; LT, low-level WM training group; CG, control group.*

### Procedure

Participants were screened for inclusion criteria via telephone and during initial personal contact. The baseline assessment consisted of various neuropsychological tests that were administered in a predefined order, balancing cognitive demands as well as computer and paper–pencil exercises. The subjects were then randomized into one of three conditions: (a) high-level WM training group (HT; *WOME* intervention), (b) low-level WM training group (LT; active control group), or (c) passive control group (CG; no contact). Participants were randomized with equal probability and stratified by sex by using the online software Research Randomizer (Urbaniak and Plous, 2013). Group allocation was concealed in envelopes and not revealed until the end of the pretest. The final groups did not differ significantly with respect to gender distribution, age, education, medication, subjective mood, functioning in everyday life, and initial WM performance (see **Table 1**).

Overall, both training groups practiced twelve sessions of 45 min of their respective training, taking part in supervised training sessions three times a week within 4 weeks. Following the training phase, a questionnaire was provided to evaluate the training, task strategies, and subjective training effects in everyday life. All participants undertook individual neuropsychological assessments at three time points: at baseline 1 week before the training phase, post-treatment within 1 week after the end of the training, and at a 3-month follow-up after training completion. If possible, parallel versions of standardized tests were used to minimize practice effects. The neuropsychological assessments lasted approximately two and a half hours.

To ensure a double-blind procedure of all participants in the training groups, the supervision of training sessions and the execution of neuropsychological assessments was carried out by different staff and in different locations: the testing took place at the Max Planck Institute for Human Cognitive and Brain Sciences Leipzig, whereas the training sessions were conducted nearby at the Clinic of Cognitive Neurology, University of Leipzig. The subjects in the training groups were told that multiple programs were evaluated in their efficacy to enhance cognitive performance.

**FIGURE 1:**
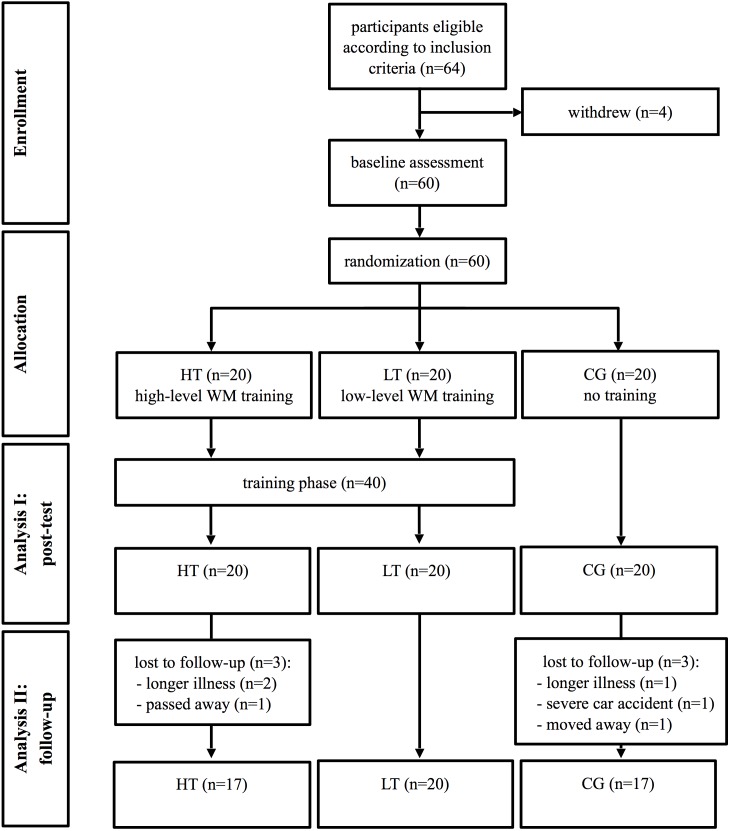
Flow chart of the study design. WM, working memory; HT, high-level WM training group; LT, low-level WM training group; CG, control group.

Of the 64 subjects that were eligible, 60 were willing to take part in the study and completed the neuropsychological baseline assessment. All training participants completed their schedules successfully and everyone returned for the post-test. In the follow-up assessment six subjects were absent: two subjects missed it due to a long period of illness, one moved away, one had a car accident and suffered severe traumatic brain injury, and two passed away. **Figure 1** presents the study design including the flow of participants from baseline to the follow-up assessment.

### Outcome Measures

Effectiveness of cognitive training was investigated on the basis of five sets of measures: (1) WM functioning was analyzed using multiple standardized neuropsychological tests to target different components of WM. (2) Other cognitive functions that partially rely on WM and could be influenced by changes in the WM system were examined. (3) To evaluate the specificity of the training, a non-target measure (reaction time) was included. (4) Questionnaires were utilized to survey the consequences of WM training in everyday life. (5) To ensure that changes were not based on unintended factors, questionnaires for various control measures, for example, depressive mood, were added. For an overview of the applied neuropsychological test battery, see **Table 2**.

**Table 2:** Applied neuropsychological test battery.

WM functioning	Cognitive functions that require WM	Everyday life functions	Non-target outcome
Digit Span (forward)	Stroop	CFQ	TAP Alertness (reaction time)
Digit Span (backward)		FEAG	
Span Board (forward)	TAP Go-NoGo	Own questionnaire	
Span Board (backward)	TMT A/B		
Spatial Addition	TAP Mental Flexibility		
Symbol Span	LPS-3		
TAP n-back			
PASAT (ISI 3/2 s)	VLMT		
Operation Span			


*WM, working memory; TAP, Test for Attentional Performance; PASAT, Paced Auditory Serial Addition task; TMT, Trail Making Test; LPS-3, Subtest 3 of the German intelligence battery “Leistungsprüfsystem”; VLMT, German version of the Auditory Verbal Learning Test; CFQ, Canadian Failure Questionnaire; FEAG, Inventory of Memory Experiences*.

#### WM Functioning

##### Digit Spans and Span Board tasks

Verbal and visuo-spatial WM spans were selected from the German Wechsler Memory Scale revised (WMS-R; Härting et al., 2004). In the Digit Span task, a series of digits were presented that the participant had to repeat immediately, forward or in reversed order. In the Span Board task, the examiner tapped on blocks that were placed irregularly on a board and the participant had to repeat the sequence forward or backward. Span lengths increased successively until the participant failed both trials of a given length. Dependent variables were the number of correct trials. A recent meta-analysis (Weicker et al., 2016) showed that the Span Board task backward is the most sensitive variable to assess changes in WM, therefore it was chosen as a criterion task.

##### Spatial Addition

The Spatial Addition is a subtest from the Wechsler Memory Scale IV (WMS-IV) that assesses visual-spatial WM (Petermann and Lepach, 2012). The examiner sequentially presented two grids filled with blue and red circles. Participants had to remember the positions of the circles and replace them with different colored circles according to a set of rules. Task difficulty increased successively, and one point was given for each correct trial. The task was stopped after producing three failed patterns. The dependent variable was the number of correct trials.

##### Symbol Span

The Symbol Span is a subtest from the WMS-IV measuring visual short-term memory. The examiner briefly displayed abstract symbols on a page that the participant had to recognize from a larger array of symbols and consider the correct order. Two points were awarded for each correct trial, one point was given if the elements but not the order matched. Task difficulty increased until five trials were answered incorrectly. The dependent variable was the total number of points.

##### N-back task

The subtest WM of the computerized Test for Attentional Performance (TAP) was used to measure updating and central executive processes of WM (Zimmermann and Fimm, 2007). Double-digits were presented one at a time, and participants had to press a button as soon as the current number matched a number that was presented two items prior. The dependent variable was the number of errors.

##### The Paced Auditory Serial Addition task (PASAT)

The Paced Auditory Serial Addition task (PASAT) measures continuous updating of information held in WM (Gronwall, 1977). The participant is instructed to listen to sequentially presented digits and add the current one to the preceding digit. Each correct calculation was rewarded with a point. The test was done twice, one trial with an inter-stimulus interval (ISI) of 3 s, and another one with an ISI of 2 s. The dependent variables were the number of correct answers.

##### Operation Span task

The Operation Span task stresses the process component of WM by introducing a secondary task (Turner and Engle, 1989; Unsworth et al., 2005). A simple mathematical equation was presented on a computer screen, and participants had to decide whether it was right or wrong (e.g., “2 × 5 - 1 = 8”). Immediately after this, a letter was presented which had to be remembered. Subsequently, the next equation was shown. The number of sequences increased successively from two to seven. The dependent variable was the number of correctly recalled letter sequences. To ensure that attention was paid to both tasks, only trails with ≥75% correct answers in the mathematical tasks were included in the analyses.

#### Cognitive Functions That Require WM

##### Executive functioning

###### Stroop task

The Stroop task is a measure of conflict resolution that requires inhibition of an over-learned response (Stroop, 1935). Participants were first asked to read aloud a list of color words (BLUE, GREEN, RED, and YELLOW) as fast as possible. After this, they were instructed to label the color in which the words are printed, hence experiencing interference of word and color name (e.g., RED written in blue). The dependent variable was the time needed for color naming.

###### Go-NoGo task

The Go-NoGo Task from the TAP measures response inhibition. On a screen, the symbols × and + were presented alternately in random order. Participants had to press a button immediately when an × appeared, but suppress their reaction when a + was shown. The outcome variable was the total number of errors.

###### Trail Making Test

The Trail Making Test (TMT) is an instrument for visuomotor processing speed and cognitive flexibility, which consists of two parts (Reitan, 1958). Part A required the participants to connect numbered circles in ascending order. In part B, numbers and letters were presented; both had to be linked alternately in ascending (numbers) and alphabetic (letters) order. The dependent variable was the ratio of time needed for both parts (TMT A - TMT B/TMT A).

###### TAP mental flexibility

The ability to switch quickly between different concepts was examined using the subtest Mental Flexibility from the TAP. A letter and number were presented simultaneously on random sides of a screen. Participants were required to press a button alternately on the side where the letter appeared, then on the side where the number appeared. The outcome variable was the total number of errors.

##### Logical reasoning

Subtest three of the German intelligence battery “Leistungsprüfsystem” (LPS-3) was used to examine logical reasoning (Horn, 1983). On a sheet of paper a series of abstract symbols were shown. Each row was constructed according to a certain rule that had to be identified. Participants had 5 min to cancel the symbols that didn’t fit to the respective rule. The outcome variable was the number of correct items.

##### Long-term memory

The German version of the Rey Auditory Verbal Learning Test (AVLT) is a word list recall test (Rey, 1941; Helmstaedter et al., 2001). A fixed sequence of 15 nouns was vocally presented five times, and participants had to repeat all remembered items after each trial. The dependent variable was the sum of correct words in this learning phase.

#### Non-target Outcome

##### TAP Alertness

Reaction time was measured using the subtest Alertness of the TAP. The task required participants to tap a button as fast as possible every time a cross appeared on a screen. The task differentiated between tonic alertness, which is the ability to generally maintain a high level of responsiveness, and phasic alertness, which is the immediate allocation of resources after the presentation of an audio warning to process an expected stimulus. The dependent variable was the mean reaction time of tonic and phasic alertness.

#### Everyday Life Functioning

##### Self-rating questionnaires

Currently, no German questionnaires are available that assess WM-related difficulties in everyday life. Hence, questionnaires were selected that largely overlap with WM demands. We used the German version of the Canadian Failure Questionnaire (CFQ), which is a self-rating scale for the assessment of cognitive failures in everyday life (Broadbent et al., 1982; Klumb, 1995); and the German version of the Inventory of Memory Experiences (FEAG) that considers memory impairments (Holzapfel, 1990). In both questionnaires, participants had to rank statements concerning everyday life memory performance on a 5-point Likert scale. The final scores were the sum of all items. Additionally, we asked the participants whether they felt any changes in everyday life performance as a result of the training. If participants answered yes, they were requested to list activities or situations where they had experienced improvements in daily life.

#### Control Measures

Depressive mood was screened with the Beck Depression Inventory (BDI; Hautzinger et al., 1995), which is based on 21 multiple choice self-reports of the severity of depressive symptoms. The sum of selected items represented the outcome variable, with scores lower than 18 indicating no acute clinical depression. After the training was completed, participants gave detailed feedback on the intervention with respect to enjoyment, motivation, subjective demands, applied strategies, previous experiences, etc.

### Intervention

**FIGURE 2:**
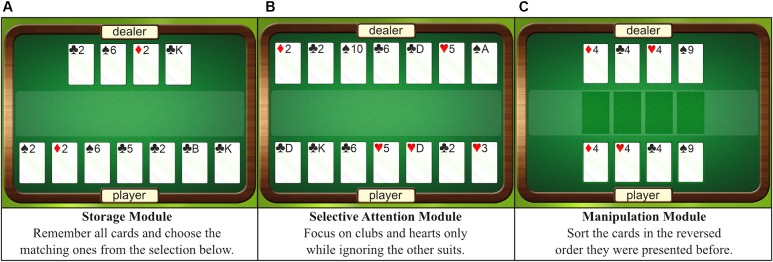
Graphical interface of the intervention. **(A–C)** Show the various modules with their respective instructions that were trained in the intervention group. During the actual training, the dealer shows several cards, which are turned over after 1 s. Here, the cards are illustrated overtly for the purpose of explanation.

The training sessions were held in a quiet room in small groups, each with a maximum of five persons and each person working at an individual computer. *WOME* consists of three hierarchically ordered modules that are designed to exercise the main components of WM on the basis of a card game: storage systems (maintenance of information), selective attention (memorizing selective parts of information and inhibiting others), and central executive/manipulation processes (active operating with the content retained in WM). For an illustration and detailed explanation of each task, see **Figure 2**. The computer-based format enabled automated and continuous adjustment of difficulty depending on the individual’s performance by modifying a range of fine-tuned parameters: presence and number of distracting stimuli (irrelevant cards on the player’s side), appearance of visual distractors (irritating illustrations on the cards surface), occurrence of distractors between encoding and response (animated animals that “walk” over the gaming table following the presentation of cards), and many more. The control training was constructed as low-level WM training. Concretely, framing conditions and stimuli were identical to the intervention, but the level of difficulty essentially stayed the same to minimize the involvement of the WM system. Motivation was kept high enhancing the level number, giving continuous feedback, and by instructing participants to react as fast as possible while avoiding slips.

### Statistical Analyses

Quantitative analyses were conducted for subjects’ raw scores in neuropsychological tests and standardized questionnaires to evaluate the effectiveness of the intervention. All raw data are provided in the **Supplementary Files**. Baseline performances of HT, LT, and CG were inspected with separate one-way analyses of variances (ANOVA). Changes over time were analyzed with a repeated measurements ANOVA model with the between-subjects factor condition (HT, LT, and CG) and within-subjects factors time point of assessment (performance at baseline, post training, and follow-up): (1) training-related improvements were inspected by comparing baseline and immediate post-training performance, (2) stability of improvements was examined by including baseline, post-test and follow-up measurements in the analyses. To investigate the impact of the intervention on comprehensive cognitive functions, performance was evaluated with respect to composite scores of multiple tests. Raw scores were converted in standardized *z*-scores to make the separate scoring systems comparable. Immediate transfer effects were calculated by subtracting the standardized *z*-scores of the pretest from the post-test. Long-term transfer effects were determined by subtracting the standardized *z*-scores of the pretest from follow-up test.

Composite scores were formed based (a) on pre-defined clusters with respect to common psychological constructs, and (b) on statistically derived data-based clusters. Psychological constructs referred to WM functioning (considering all WM tests or exclusively span tests), cognitive functions that require WM (in general as well as executive functions, logical reasoning, and long-term memory), and everyday life functioning (standardized questionnaires). Data-based clusters of WM tests were obtained using principal component analysis with oblique rotation (direct oblimin, missing values replaced with means). The Kaiser–Meyer–Olkin measure confirmed the sampling adequacy for the analysis, KMO = 0.73. The initial analysis revealed three components with eigenvalues over Kaiser’s criterion of 1 that in combination explained 61% of the variance. The first component was composed of the Span Board tasks, both versions of the PASAT, and the n-back task; the second component included the Digit Span task forward and the Operation Span task; and the third component consisted of the Symbol Span, Spatial Addition, and Digit Span task backward.

Analyses were carried out with the Statistical Package for Social Sciences SPSS, version 22 (IBM Corp, 2013). The overall significance level was set to *p* < 0.05 (two-tailed). For all ANOVAs concerning intervention effects, we applied false discovery rate (FDR, *q* = 0.20) to correct for multiple comparisons in consideration of the large test battery. FDR has been shown to be an adequate method to preserve power when sample size is limited (Benjamini and Hochberg, 1995). Interactions were decomposed conducting *post hoc t* tests with Bonferroni’s correction for multiple comparisons. An effect size estimate for describing the proportion of variability represented by independent factors is given by partial eta-squared ($\eta_{p}^{2}$), with $\eta_{p}^{2}$ ≥ 0.01 indicating a small effect, $\eta_{p}^{2}$ ≥ 0.06 a moderate effect, and $\eta_{p}^{2}$ ≥ 0.14 a large effect; effect sizes for differences between two conditions and dependent variables are specified by Cohens’s *d*, with *d* ≥ 0.2 indicating a small effect, *d* ≥ 0.5 a moderate effect, and *d* ≥ 0.8 a large effect (Cohen, 1988).

## Results

### Training Benefits

#### Improvement in the Training Task

**FIGURE 3:**
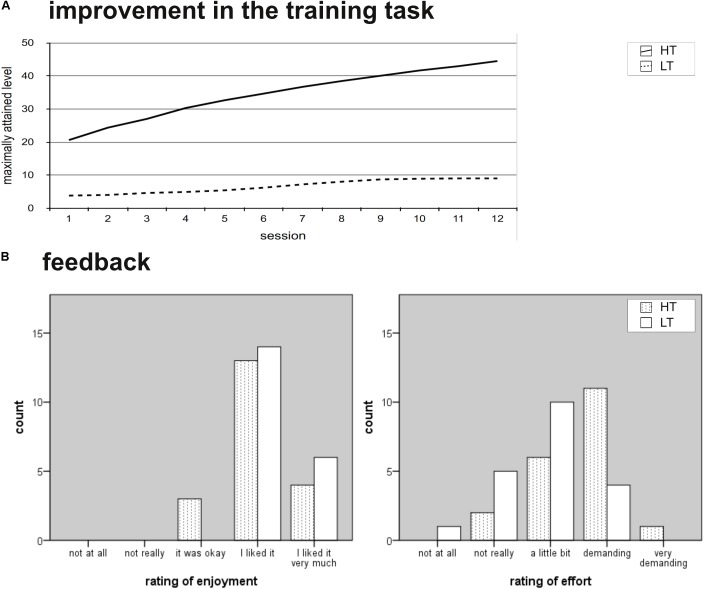
Training benefits. WM, working memeory; HT, high-level WM training group; LT, low-level WM training group. **(A)** Illustrates the performance in the trained tasks for each of the 12 training sessions. The lines show the mean level for each session per group. Note that the level structure of the low-level training program was designed to be limited in progression. **(B)** Illustrates the feedback given from HT and LT regarding enjoyment and subjective effort.

**Figure 3A** illustrates the improvements of HT and LT in the trained task. HT showed continuous progression of the mean level of difficulty from the first to last training session, *t*(19) = 16.32, *p* < 0.001, *d* = 1.29. Although individual task performance varied between subjects, nobody showed signs of a ceiling effect after twelve sessions of training [e.g., mean improvement from 11th to 12th training session: *t*(19) = 6.94, *p* < 0.001, *d* = 0.20]. Progress in the trained task was limited per definition in LT. Training success in HT was predictable by performance at the initial session [β = 0.54, *t*(19) = 2.75, *p* = 0.013], as well as by WM functioning at baseline [e.g., Digit Span backward: β = 0.63, *t*(19) = 3.44, *p* = 0.003, spatial addition: β = .68, *t*(19) = 3.94, *p* = 0.001], arguing for the validity of the training. Moreover, the correlations between the initial performance in the trained task and WM outcome measures support the hypothesis that they tap overlapping processes and should improve following the intervention.

#### Feedback

Both training conditions received comparable positive feedback. Training was rated as enjoyable (HT: *M* = 4.05, *SD* = 0.61, LT: *M* = 4.30, *SD* = 0.47; difference according to Median Test *p* = 0.715) and both groups were equally motivated (*M* = 4.10, *SD* = 0.45, and *M* = 4.15, *SD* = 0.49, respectively; *p* = 1.00). Nevertheless, training difficulty was judged differently, with the adaptive WM training program evaluated as more demanding than the low-level control training program (HT: *M* = 3.35, *SD* = 0.76, LT: *M* = 2.85, *SD* = 0.81; *p* = 0.024). The groups did not differ with regard to prior experience with card games (HT: *M* = 1.61, *SD* = 0.92, LT: *M* = 2.25, *SD* = 0.40; *p* = 0.718) or computers (HT: *M* = 4.61, *SD* = 0.98, LT: *M* = 3.80, *SD* = 1.47; *p* = 0.957). Altogether, the manipulation check indicated that the blinding procedure was successful: The WM training as well as the control training was accepted and diligently completed, with the only difference being the perceived difficulty of the applied tasks (see **Figure 3B**).

### Immediate Transfer Effects

No significant performance differences between the conditions were found at pretest assessment [except for the Span Board task forward, *F*(2,57) = 4.17, *p* = 0.02, $\eta_{p}^{2}$ = 0.13, where LT outperformed HT: *t*(38) = 2.78, *p* = 0.008, *d* = 0.88].

**FIGURE 4:**
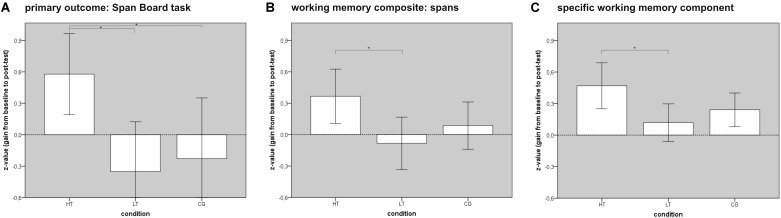
Effect of *WOME* WM training. WM, working memory; HT, high-level WM training group; LT, low-level WM training group; CG, control group. The bars show significant gains from baseline to post-test in WM function in the intervention group. Error bars represent ± 2 standard errors. Significant group differences are marked with an asterisk. **(A)** Illustrates the improvement in the primary outcome variable (Span Board task backward), **(B)** shows the improvement in a WM composite score (Digit Spans and Span Board tasks), **(C)** illustrates the improvement in the first WM component of the multiple component analysis (composed of Span Board, PASAT, and n-back tasks).

After training, near transfer effects on untrained WM tasks were found on several levels:

(1) Considering single test results of the primary outcome variable, there was a significant improvement in the Span Board task backward [significant time × condition interaction: *F*(2,57) = 4.38, *p* = 0.017, $\eta_{p}^{2}$ = 0.13; no significant main effect of time: *F*(1,57) = 1.84, *p* = 0.180, $\eta_{p}^{2}$ = 0.03; no significant main effect of condition: *F*(2,57) = 0.54, *p* = 0.536, $\eta_{p}^{2}$ = 0.02; see **Figure 4A**]. *Post hoc t*-tests with a Bonferroni adjusted alpha level of 0.017 per test (0.05/3) revealed a significant improvement from baseline to post-test for HT, *t*(19) = 4.06, *p* = 0.001, *d* = 0.88, whereas the other groups did not show any changes [LT: *t*(19) = 0.71, *p* = 0.489, *d* = 0.16, CG: *t*(19) = 0.12, *p* = 0.909, *d* = 0.18]. With respect to the other WM tests, no significant time × condition interactions were found.

(2) Regarding composite scores based on neuropsychological constructs, a significant intervention effect was shown on a composite score of different WM spans [*F*(2,57) = 3.42, *p* = 0.040, $\eta_{p}^{2}$ = 0.11; see **Figure 4B**]. *Post hoc t*-tests with a Bonferroni adjusted alpha level of 0.017 per test (0.05/3) showed a significantly higher gain for HT compared to LT [*t*(38) = 2.50, *p* = 0.017, *d* = 0.79], but not compared to CG [*t*(38) = 1.63, *p* = 0.112, *d* = 0.52]. No difference between LT and CG was found [*t*(38) = 1.01, *p* = 0.321, *d* = 0.32]. No significant interaction was found when all applied WM tests were combined into a general WM functioning score [*F*(2,57) = 2.38, *p* = 0.102, $\eta_{p}^{2}$ = 0.08]; however, a closer inspection of the subgroups revealed a significantly larger gain for HT compared to LT [*t*(38) = 2.06, *p* = 0.046, *d* = 0.65], which was not the case for other direct comparisons [HT vs. CG: *t*(38) = 0.83, *p* = 0.415, *d* = 0.26; difference between LT and CG: *t*(38) = 1.46, *p* = 0.153, *d* = 0.46].

(3) The first WM component of the multiple component analysis (composed of Span Board, PASAT, and n-back tasks) was significantly influenced by the intervention [*F*(2,57) = 3.62, *p* = 0.033, $\eta_{p}^{2}$ = 0.11; see **Figure 4C**]. Pairwise comparisons showed a significant advantage of HT over LT with a moderate to large effect size [*t*(38) = 2.49, *p* = 0.017, *d* = 0.79], but not over CG [*t*(38) = 1.69, *p* = 0.100, *d* = 0.53], according to Bonferroni’s adjusted alpha level of 0.017 per test (0.05/3). LT and CG showed similar gains [*t*(38) = 1.03, *p* = 0.312, *d* = 0.33]. The other WM components were not affected by the intervention [second component: *F*(2,56) = 0.31, *p* = 0.736, $\eta_{p}^{2}$ = 0.01; third component: *F*(2,57) = 0.62, *p* = 0.540, $\eta_{p}^{2}$ = 0.02].

In contrast to the significant effects on near transfer measures of WM, no far transfer on cognitive functions that require WM was found [*F*(2,55) = 0.21, *p* = 0.812, $\eta_{p}^{2}$ = 0.01].

**FIGURE 5:**
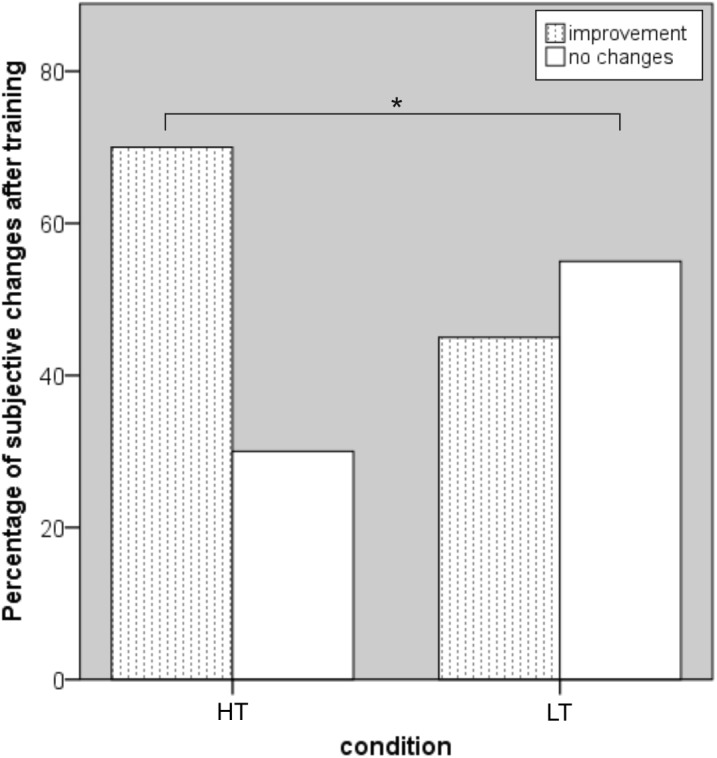
Subjective evaluation of improvements in everyday life functions. WM, working memory; HT, high-level WM training group; WM, working memory; LT, low-level WM training group. HT significantly outperformed LT by reporting more positive changes in daily life performance (significant effect marked with an asterisk). Note that both training groups were blind with respect to their condition.

Individual evaluation of everyday life functioning indicated substantial changes in favor of HT [χ^2^(1, *N* = 40) = 12.79, *p* < 0.001; illustrated in **Figure 5**]. In particular, subjects reported improved memorization of shopping lists, names, telephone numbers, and vocabularies in foreign language acquisition, as well as enhanced attention and navigation while driving. These effects, however, were not found on standardized questionnaires of everyday life performance [*F*(2,49) = 0.28, *p* = 0.759, $\eta_{p}^{2}$ = 0.01].

No changes were found in the non-target outcome [mean reaction time, *F*(2,56) = 0.46, *p* = 0.635, $\eta_{p}^{2}$ = 0.01], demonstrating the specificity of the WM training.

### Long-Term Maintenance of Transfer Effects

The near transfer effects on untrained WM tasks that were seen immediately after the end of training were not maintained over a 3-month follow-up period. A trend for stability was seen in the Span Board task backward [*F*(4,102) = 2.44, *p* = 0.052, $\eta_{p}^{2}$ = 0.09] but vanished for the training gain regarding the combined score of all WM span tests [*F*(4,102) = 1.72, *p* = 0.152, $\eta_{p}^{2}$ = 0.06] and the initially affected WM component [*F*(4,102) = 1.52, *p* = 0.202, $\eta_{p}^{2}$ = 0.06]. For detailed information regarding means and standard deviations of all neuropsychological outcomes, see supporting information (**Supplementary Table S1**).

## Discussion

There is an intensive need for effective and scientifically evaluated cognitive intervention programs in order to meet the demands of our increasingly aging society. The present study evaluated the feasibility and effectiveness of *WOME*, a new theory-based WM training program, by implementing a randomized, placebo-controlled, and double-blind trial in healthy older adults, featuring: (1) a high-level WM training group (HT) that received the new intervention, (2) a low-level WM training group (LT) that represented an active control condition, and (3) a passive control group (CG). The results of our study suggest that WM training can indeed enhance specific cognitive functioning, that is, WM, in an older population. Improvement in WM function was demonstrated on enhanced performance in non-trained near transfer tasks with moderate to large effect sizes, shown on a single test level (span board task backward), and in two different composite scores (data-driven cluster of WM tests, and theoretically motivated construct of WM functioning, i.e., span measures). What may be highly relevant is that subjects reported a positive impact on everyday life (e.g., better able to memorize telephone numbers, shopping lists, and improved concentration). We showed that the intervention effects were specific for the WM system and that there was no transfer to other cognitive functions. Furthermore, the effects were short-term rather than stable, being substantially diminished by the 3-month follow-up with only little evidence suggesting long-term maintenance of WM training.

The result that WM training produces enhanced functioning of the WM system, observed in performance gains in tasks more or less similar to the trained ones, is in line with recent meta-analyses (Karbach and Verhaeghen, 2014; Melby-Lervag et al., 2016; Weicker et al., 2016; Soveri et al., 2017), thus strengthening the evidence that training WM has the potential to improve WM function, even in older subjects. Although the majority of subjects declared that they used some kind of strategy, the observed transfer to untrained WM tasks suggests that domain-general mechanisms in terms of a core training program were promoted by *WOME* (Morrison and Chein, 2011). The mechanism of such transfer effects is still unknown. Several studies have proposed that shared cognitive processes (e.g., updating) and neuronal substrates enable transfer from trained to untrained tasks (Dahlin et al., 2008; Salminen et al., 2012; Harrison et al., 2013; Beatty et al., 2015; Waris et al., 2015). In our data, the idea that WM training and outcome measures tap overlapping processes was supported by correlations between the initial performance in the trained task and various WM outcome measures. Other hypotheses state that increased attention control is responsible for performance changes, which is supported by studies that showed enhanced WM performance after training of selective attention (Shin et al., 2015; Greenwood and Parasuraman, 2016; Schmicker et al., 2016), and that WM is not enhanced by capacity but by efficiency training (von Bastian and Oberauer, 2014). To date, it is assumed that WM training does induce some kind of change in the underlying cognitive system, but we do not yet understand exactly what these are.

Despite several single studies indicating widespread transfer to other cognitive functions in older populations (Borella et al., 2010, 2013; Brehmer et al., 2012), our findings agree with more rigorous meta-analyses accounting for methodological issues that transfer effects which go beyond WM are non-existent or very small at older ages (Redick et al., 2015; Schwaighofer et al., 2015; Melby-Lervag et al., 2016; Soveri et al., 2017). The absence of far transfer effects in the present study could be rooted in the high specificity of the stimuli and the limited diverseness of the training tasks which may have reduced overlapping processes of WM and, for example, executive functions. Training alternately various components of WM may also have induced adverse effects. Possibly, efficient training of selective attention processes was interrupted by including sessions of (unnecessary) storage or manipulation training (cf. Greenwood and Parasuraman, 2016; Schmicker et al., 2016). Only a few studies explored clinical or everyday relevance in older populations with promising, yet mixed results (Brehmer et al., 2012; McAvinue et al., 2013; Cantarella et al., 2017). Our study fits in as we have found a perceived positive impact on everyday life in HT compared to LT, but there were no differences in standardized questionnaires on memory and attention performance. A possible explanation is that questionnaires on cognitive functioning are conceptualized to identify trait rather than state measures (Bridger et al., 2013) and may therefore be less sensitive for changes within a short period of time, whereas self-reports have been shown to reveal rapid alterations (Mulligan et al., 2017).

In contrast to previous studies (Dahlin et al., 2008; Li et al., 2008; Borella et al., 2010; Richmond et al., 2011), the intervention delivered only limited evidence for sustained training effects (but see Buschkühl et al., 2008, for similar results). This raises the question of whether there are special features of interventions that are able to produce long-term effects. One crucial predictor already identified for long-lasting efficacy is the amount of training (Karbach and Verhaeghen, 2014; Weicker et al., 2016). More than 20 sessions seem to be necessary in order to induce long-term effects (Thöne-Otto, 2017). Bearing this in mind, our intervention of 12 sessions may have been too short. An indication for this hypothesis is provided in the analysis of course of performance on the trained task, showing no asymptotic course or ceiling effect of the individual’s performance, a typical trajectory found by the end of a learning task (Ritter and Schooler, 2001).

Other moderators that are discussed to influence training efficacy include motivation (Jaeggi et al., 2014; Au et al., 2015), self-perceived stress (Leung et al., 2016), initial cognitive capacities (Basak and Zelinski, 2013; Holmes and Gathercole, 2014; Titz and Karbach, 2014; Weicker et al., 2016; Borella et al., 2017), and personality measures (Studer-Luethi et al., 2012; Alesi et al., 2015; Minear et al., 2016). To account for such differences, we did not only include a passive control group, but also an active condition to check for various placebo effects. By setting comparable time schedules, training conditions, and a double-blind procedure, we ensured that both training groups received the same amount of care from the staff and were equally involved in the study. Despite that LT performed an intervention with low-level difficulty overall, the level description numbers increased in a comparable way to HT, providing feedback and perseverative motivation for the individuals. The procedure was successful because both groups responded similarly in rating the motivation and enjoyment of their intervention; it was exclusively the perceived effort and demand on the WM system that separated the conditions. The descriptive analysis of the data of CG showed that different experiences can lead to result patterns that are difficult to understand and hinder comparisons between conditions. Therefore, we agree with leading researchers in the field who urged future studies to always provide comparable placebo-controlled conditions (Shipstead et al., 2012; Dougherty et al., 2016; Melby-Lervag et al., 2016; Weicker et al., 2016).

Redick et al. (2015) recommended seven methodological criteria for the evaluation of WM trainings to produce valid and reliable results: (1) use of an active control group, (2) sufficient sample size, (3) use of objective measures and double-blind study design, (4) evidence for positive transfer results to WM, (5) transfer results follow a sensible pattern, (6) follow-up transfer assessment, and (7) multiple measures of each construct. Although we accurately implemented each element, there are several limitations regarding the interpretation of the results. The first and most obvious issue refers to the relatively small sample size compared to large-scale evaluations. The achieved power computed *post hoc* for our study was 0.74 at the post-test and 0.66 and follow-up measurement given an estimated effect size of *g* = 0.60 for immediate near transfer and *g* = 0.54 for long-term maintenance in healthy older adults (Weicker et al., 2016). Hence, there was a chance of around 70% of observing a near transfer effect after WM training. The realization of intervention designs with larger sample sizes is difficult to conduct in a local setting, and, consequently, implies either home-based training or a multi-center application—which we explicitly avoided by focusing on the control of side effects provoked by various surrounding conditions (Lampit et al., 2014). Beyond traditional significance testing and power analyses, the observed effect sizes were moderate to large, which confirms the assumption that a meaningful improvement was achieved after WM training. The observed moderate effect size regarding our criterion task in the follow-up test revealed indication that efficacy of WM training might last longer than proposed by null-hypotheses testing. Another limitation refers to the critical analysis of the descriptive data; transfer results should follow a sensible pattern that consists of similar results of all conditions at pretest and a greater improvement of the intervention group compared to the control group at post-test. The requested pattern is observed in most outcomes, but our criterion task fails to demonstrate this (there was an improvement in HT, while both LT and CG showed stable or slightly decreased performances). Hence, in addition to the conclusion that WM training resulted in improved WM function, measured by the span board task backward, critical alternatives refer to sampling errors or regression toward the mean (Moreau et al., 2016). By demonstrating the effect not only in a single test, but also on latent factors, we expect the observed interaction effects to be justified as improvements in WM function (Ackerman et al., 2005).

## Conclusion

The *WOME* is a new, specific and theory-based WM training program that efficiently improves WM function, and there is some indication that it has a relevant effect on everyday life. To date, there is little evidence that benefits are long lasting, so continuous or intermittent training sessions are highly recommended. By finding no evidence of transfer effects to other cognitive domains, we join rather skeptical researchers in the field in assuming that WM training represents a specific intervention targeting WM function, and that it is neither a panacea for various cognitive functions nor a key for slowing down cognitive decline in general. It is, however, quite conceivable that continuous training contributes to a deceleration of cognitive decline with respect to WM. Numerous studies have shown that the brain remains modifiable across one’s lifespan and that even old-old adults are able to benefit from an enriched environment with the potential to improve cognitive performance (Kramer and Willis, 2002; Greenwood, 2007; Mora et al., 2007). If interventions manage to stabilize WM performance over several years, we should pursue this approach to allow people to live as high functioning individuals for as long as possible. The challenge of future research is to detect mechanisms that provide the best transfer effects on cognitive functions and, more importantly, on everyday life by methodologically solid designs that account for a manageable feasibility/cost–benefit ratio. Much work is still needed regarding variability in older populations and individual differences (genetic predispositions, lifestyle, physical and mental activity) to discover moderators of resilience in aging and understand their impact on the plasticity of cognitive functions.

## Data Availability

All datasets collected and analyzed for this study are included in the manuscript and the **Supplementary Files**.

## Author Contributions

JW, SF, JL, KM, AV, and AT-O substantially contributed to conception and design of the study. JW and NH organized and performed the experiments, collected the data, and performed the statistical analyses. JW wrote the first draft of the manuscript. All authors discussed the results and contributed to manuscript revision.

## Conflict of Interest Statement

Hasomed GmbH funded research assistants for the study, supported the technical implementation of the intervention and provided it free of charge for the purposes of this research. Since March 2017, JW has been employed at Hasomed GmbH in the context of a new research project. The remaining authors declare that the research was conducted in the absence of any commercial or financial relationships that could be construed as a potential conflict of interest.
