# Supplementary material for: WOME: Theory-Based Working Memory Training — A Placebo-Controlled, Double-Blind Evaluation in Older Adults
Source: Front Aging Neurosci. 2018 Aug 14;10:247. doi: 10.3389/fnagi.2018.00247 (PMC6102413; doi:10.3389/fnagi.2018.00247)
Supplement: Supplementary file 1 [file Table_1.PDF]

Supplemental Material for *WOME: Theory-based working memory training. A placebo-controlled, double-blind evaluation in older adults*.
Weicker, Hudl, Frisch, Lepsien, Mueller, Villringer, & Thöne-Otto (2018)

Table S1 Mean performance and standard deviations of the neuropsychological outcomes.

outcomes	WOME WM training group			active control group			passive control group		
	T1	T2	T3	T1	T2	T3	T1	T2	T3
**WM functions**									
Digit Span (forwards)	6.90<br>(1.52)	7.70<br>(1.72)	7.35<br>(1.66)	7.00<br>(1.08)	7.05<br>(1.73)	7.20<br>(1.44)	7.30<br>(1.53)	7.55<br>(1.73)	7.47<br>(2.15)
Digit Span (backwards)	6.75<br>(1.68)	6.65<br>(1.76)	7.06<br>(1.48)	5.85<br>(1.46)	5.95<br>(1.64)	6.20<br>(1.51)	6.25<br>(1.37)	6.55<br>(1.57)	7.06<br>(1.71)
Span Board (forwards)	7.20<br>(1.11)	7.45<br>(1.70)	7.76<br>(2.02)	8.30<br>(1.38)	7.95<br>(1.28)	8.75<br>(1.41)	7.95<br>(1.19)	7.95<br>(1.88)	8.88<br>(1.65)
Span Board (backwards)	6.55<br>(1.10)	7.70<br>(1.46)	7.12<br>(1.54)	7.65<br>(1.67)	7.40<br>(1.54)	7.70<br>(2.00)	7.20<br>(1.61)	7.15<br>(1.39)	8.00<br>(1.46)
Spatial Addition	10.25<br>(2.12)	11.75<br>(2.85)	11.88<br>(3.46)	10.90<br>(2.77)	12.00<br>(2.97)	12.45<br>(2.80)	11.05<br>(3.55)	11.80<br>(3.65)	12.59<br>(3.00)
Symbol Span	20.00<br>(5.18)	21.25<br>(4.63)	24.82<br>(6.91)	18.50<br>(5.32)	20.85<br>(4.52)	21.85<br>(4.56)	18.00<br>(4.85)	22.85<br>(5.33)	22.47<br>(5.90)
N-back	5.40<br>(9.37)	2.35<br>(2.93)	2.88<br>(5.41)	3.80<br>(4.63)	3.55<br>(5.33)	2.40<br>(2.33)	6.45<br>(9.45)	2.05<br>(3.40)	2.24<br>(3.93)
PASAT (ISI 3 sec)	41.45<br>(11.55)	46.95<br>(9.32)	48.18<br>(7.43)	42.20<br>(11.02)	47.30<br>(9.84)	45.65<br>(10.33)	44.40<br>(10.51)	49.35<br>(9.00)	50.00<br>(8.63)
PASAT (ISI 2 sec)	30.25<br>(9.81)	35.20<br>(8.68)	38.53<br>(6.86)	29.05<br>(7.35)	34.10<br>(8.78)	33.65<br>(9.34)	34.30<br>(10.86)	36.60<br>(11.08)	39.88<br>(9.24)
**cognitive functions that require WM**									
executive functioning									
Stroop	114.53<br>(29.67)	112.74<br>(26.97)	108.65<br>(27.01)	128.20<br>(23.83)	114.80<br>(13.36)	117.30<br>(18.80)	120.40<br>(31.31)	113.20<br>(24.97)	107.38<br>(22.69)
Go-NoGo	1.10<br>(2.15)	1.00<br>(1.49)	0.41<br>(0.62)	0.50<br>(1.19)	0.90<br>(1.25)	1.20<br>(2.33)	0.80<br>(0.70)	0.80<br>(1.28)	0.47<br>(0.72)
TMT	1.28<br>(0.68)	1.25<br>(0.73)	1.10<br>(0.56)	1.17<br>(0.58)	1.14<br>(0.72)	1.50<br>(0.75)	1.27<br>(0.85)	1.22<br>(0.75)	1.11<br>(0.51)
Mental Flexibility	2.65<br>(2.89)	1.30<br>(1.87)	0.71<br>(1.11)	2.45<br>(3.49)	1.40<br>(3.24)	2.00<br>(4.70)	2.30<br>(4.14)	2.30<br>(3.59)	1.24<br>(2.71)

Supplemental Material for *WOME: Theory-based working memory training. A placebo-controlled, double-blind evaluation in older adults***.**
Weicker, Hudl, Frisch, Lepsien, Mueller, Villringer, & Thöne-Otto (2018)

outcomes	WOME WM training group			active control group			passive control group		
	T1	T2	T3	T1	T2	T3	T1	T2	T3
logic reasoning									
LPS-3	23.40<br>(4.31)	25.10<br>(6.10)	26.41<br>(4.30)	22.05<br>(4.27)	23.85<br>(4.43)	24.50<br>(4.35)	23.30<br>(4.80)	24.70<br>(4.53)	26.18<br>(4.14)
long-term memory									
VLMT	49.05<br>(9.18)	50.60<br>(7.37)	54.47<br>(7.01)	51.35<br>(6.31)	51.70<br>(7.18)	54.75<br>(7.70)	51.60<br>(8.75)	52.55<br>(9.17)	53.29<br>(9.30)
**non-target outcome**									
reaction time	264.36<br>(49.24)	264.34<br>(47.10)	280.22<br>(55.93)	260.17<br>(30.37)	266.58<br>(43.41)	276.70<br>(68.06)	255.30<br>(44.84)	262.40<br>(54.94)	266.37<br>(72.64)
**everyday life functions**									
CFQ	17.25<br>(12.31)	17.83<br>(12.30)	16.35<br>(10.57)	20.53<br>(10.51)	20.82<br>(8.88)	21.20<br>(10.12)	22.45<br>(7.07)	19.39<br>(8.71)	17.56<br>(6.96)
FEAG	27.15<br>(14.47)	27.44<br>(16.28)	26.94<br>(13.94)	27.18<br>(12.70)	27.76<br>(11.76)	29.07<br>(14.46)	29.80<br>(8.80)	28.28<br>(10.79)	27.13<br>(9.62)

Note: All values represent raw scores and refer to correct items, except for N-back, Go-NoGo and Mental Flexibility (errors), Stroop (time in sec), TMT (ratio (A – B) / A), reaction time (time in msec) and everyday life questionnaires (sum of items). N = 60 for baseline (T1) and post assessment (T2), N = 54 at 3-month follow-up (T3; WM training group (WOME) n = 17, active control group (aCtrl) n = 20, passive control group (pCtrl) n = 17), except for Stroop (WOME T1/T2: n = 19; pCtrl T3: n = 16), TMT (pCtrl T1: n = 19), reaction time (aCtrl T1: n = 19), CFQ and FEAG (WOME T2: n = 18; aCtrl T1/T2: n = 17, T3: n = 15; pCtrl T2: n = 18, T3: n= 16).
